# Membrane Distillation of Saline Water Contaminated with Oil and Surfactants

**DOI:** 10.3390/membranes11120988

**Published:** 2021-12-17

**Authors:** Wirginia Tomczak, Marek Gryta

**Affiliations:** 1 Faculty of Chemical Technology and Engineering, Bydgoszcz University of Science and Technology, 3 Seminaryjna Street, 85-326 Bydgoszcz, Poland; 2 Faculty of Chemical Technology and Engineering, West Pomeranian University of Technology in Szczecin, ul. Pułaskiego 10, 70-322 Szczecin, Poland

**Keywords:** direct contact membrane distillation, oily wastewater, polypropylene membrane, sea water, scaling, surfactant, wetting

## Abstract

Application of the membrane distillation (MD) process for the treatment of high-salinity solutions contaminated with oil and surfactants represents an interesting area of research. Therefore, the aim of this study is to investigate the effect of low-concentration surfactants in oil-contaminated high-salinity solutions on the MD process efficiency. For this purpose, hydrophobic capillary polypropylene (PP) membranes were tested during the long-term MD studies. Baltic Sea water and concentrated NaCl solutions were used as a feed. The feed water was contaminated with oil collected from bilge water and sodium dodecyl sulphate (SDS). It has been demonstrated that PP membranes were non-wetted during the separation of pure NaCl solutions over 960 h of the module exploitation. The presence of oil (100–150 mg/L) in concentrated NaCl solutions caused the adsorption of oil on the membranes surface and a decrease in the permeate flux of 30%. In turn, the presence of SDS (1.5–2.5 mg/L) in the oil-contaminated high-salinity solutions slightly accelerated the phenomenon of membrane wetting. The partial pores’ wetting accelerated the internal scaling and affected degradation of the membrane’s structure. Undoubtedly, the results obtained in the present study may have important implications for understanding the effect of low-concentration SDS on MD process efficiency.

## 1. Introduction

Membrane distillation (MD) is a separation process, where water vapor molecules and/or volatile compounds flow from a hot aqueous solution through pores of a hydrophobic membrane due to the difference in the partial pressure across a membrane, as a result of the temperature gradient [1]. In recent years, MD has gained a growing interest in research and commercial development, mainly in the field of desalination and wastewater treatment. Undoubtedly, this is because it offers technological advantages, including:

(i) Capabilities in treating high-salinity brines [2,3,4,5,6,7,8,9,10,11,12];
(ii) Low operating pressure and temperature [1,3,7,13,14,15,16];
(iii) Low capital cost [7,14,17,18];
(iv) Potential to be coupled with alternative heat sources [13,19,20,21];
(v) No extensive pretreatment of the feed [1,15];
(vi) Flexibility to be integrated with other processes and technical simplicity [1,22].

Generally, membranes used for MD process are prepared for ultrafiltration and microfiltration processes through phase inversion. Typical MD membranes are made of hydrophobic materials, mainly polypropylene (PP), polyvinylidene fluoride (PVDF), polytetrafluoroethylene (PTFE) and polyethylene (PE) [23]. So far, many researchers have made remarkable achievements in MD process development. For instance, in recent years there has been growing interest in improving the properties of the membranes’ surface via various methods, such as plasma treatment [24,25,26,27,28,29], electrospinning [30,31,32,33,34], dip coating [35,36,37] and grafting [38,39]. Moreover, great progress has been made in fabrication and application of omniphobic and Janus MD membranes [19,40,41,42,43,44,45,46]. Kalla [47], in a recently published review article, has demonstrated that the number of research papers published from the year 2000 to 2020 in the MD field increased continuously. However, although the MD process was invented in 1963 [48] and has been carried out using commercial membranes around 2006 [49], implementations at the large-scale are still limited. This is related to the fact that state-of-the-art MD, similar to other membrane separation processes, still has limitations. Indeed, contributions of numerous investigations have highlighted issues related to the MD process, such as pore wetting and membrane scaling that lead to significant reductions in the cost-effectiveness of the process [50].

Hou et al. [14] underlined that among the above-mentioned issues, membrane pore wetting is particularly challenging since it leads to a decrease in membrane performance and permeate quality. Thus, occurrence of this phenomenon prevents the continuous operation of MD processes. As has been clearly highlighted by Wang et al. [51], the membrane wetting is understood to a significantly lesser extent than the fouling phenomenon. Generally speaking, membrane pore wetting occurs when the membrane material loses its hydrophobicity and consequently, liquid water overcomes the entry pressure of the membrane pores and reaches the permeate side. Recent research has shown that this phenomenon is inevitable in the MD process and involves a complex of various physical and chemical interactions [52]. For instance, membrane wetting has been widely reported during the MD process applied for desalination of high salinity solutions [6,8,10,11,12]. Furthermore, it is well known from the literature that the wetting phenomenon is particularly intense in the course of MD processes of feeds containing low surface tension components, such surfactants and oils, that adsorb onto the membrane, making it gradually more hydrophilic [49,53,54]. Therefore, in addition to high porosity and high mechanical and chemical stability [55] MD membranes should have the following properties ensuring the prevention of water penetration: hydrophobicity, small pore size and a structure without voids [3]. Notably, mechanisms, reversal, applied aspects and prevention of membrane wetting in the MD process have been discussed in detail in several recently published review articles [13,20,52,56,57].

Nowadays, surfactants have a broad range of applications, hence, they commonly exist in saline wastewaters [14]. Indeed, they are used as major components of several products, such as detergents, cosmetics, pharmaceuticals, plastics, mining and pesticides [58,59,60,61]. In addition to the above, surfactants play a substantial role in industrial processes, such as oil recovery [62,63,64,65,66]. Typical surfactants can be divided into two types: non-ionic (e.g., Span 20) and ionic (e.g., sodium dodecyl sulfate) (SDS)) [67]. Generally, surfactants have amphiphilic molecular structure and, when dissolved in water, adsorb at the air–water interface, creating a water-soluble monolayer [68,69,70]. In membrane technology, surfactants deposition on the membrane surface may lead to reducing both the membrane surface contact angle and the feed surface tension and, consequently, membranes wetting is observed [20]. Results obtained by Chew et al. [71] have revealed that the PVDF membrane fouling and wetting behaviours are affected by surfactant concentration and hydrophobicity. In turn, Eykens et al. [72] have studied wetting resistance of membranes made of PTFE, PE and PES during the MD process of waste streams containing surfactants and oil. The Authors have shown that the limiting surfactant concentration increases with increasing hydrophobicity and oleophobicity of the membranes used. In another study, Han et al. [73] have investigated the effect of oil, SDS and salt (NaCl) on the MD process performance. For this purpose, the flat-sheet PVDF membranes have been used. The above-mentioned authors have demonstrated that the presence of SDS in the oil-in-water emulsions does not have the significant impact on the MD membranes’ performance and leads to a slight increase in the tendency for membrane wetting. Similar results have been obtained for the feed containing NaCl alone or NaCl and oil-in-water emulsions. However, it has been found that the presence of both NaCl and SDS in oil-in-water emulsions led to an increased phenomenon of membrane wetting and a significant reduction in the permeate flux. In addition, it has been demonstrated that in the absence of oil in the feed, the MD performance decreased progressively with increasing NaCl and SDS concentrations. The above-mentioned authors have concluded that further research on the effect of oil, salt and surfactant on the MD process performance is required.

This conclusion is in line with that presented by Chamani et al. [57] who have indicated that research focused on the membrane wetting is insufficient. Indeed, the authors, on the basis of the Scopus database (October 2020), showed that out of 1932 publications related to MD and desalination processes, only 206 articles investigated the wetting phenomenon. This clearly indicates that in order to be useful from practical point of view, further research focused on this issue is essential. 

**Figure 1:**
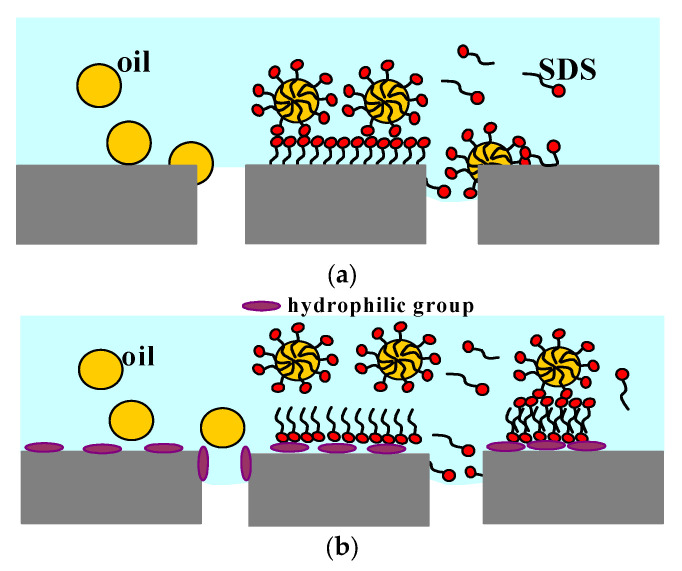
The effect of surface chemistry on the surfactant and oil absorption on the membrane surface: (**a**) new hydrophobic membrane (initial period); (**b**) MD membrane with hydrophilic group formed on the membrane surface during long-term module exploitation.

The differences in the obtained results may result from the fact that the properties of MD membranes depend not only on their structure and surface chemistry, but also on the operating time [24,25,26]. Hydrophobic polymers exhibit high chemical resistance; hence, it is commonly assumed that polymeric membranes demonstrate stable properties. Experimental tests conducted for several hours allow to determine the impact of the structural or chemical modifications of membranes on their resistance to wetting by surfactants or oils [20,34,74,75,76]. Importantly, the long-term studies confirmed the stability of the membranes’ properties during the initial period, which, usually does not exceed 100 h of MD process run [7,20,77]. On the other hand, when MD membranes are used for much longer separation processes, the observed properties differ from those found in the initial period [24,25,26,34,77,78]. For this reason, determining the properties of membranes used in industrial installations requires MD studies conducted for over 500–1000 h [78,79]. It should be noted that during long operations, as a result of the increased temperature of the feed, hydrophilic compounds are formed on the membrane surface [80]. Consequently, as shown in Figure 1, the membrane is covered with a layer of adsorbed water, which leads to changes in the membrane interactions with the surfactants and oil droplets present in the feed and membranes resistance to wetting [79].

As a result of the hydrophobic–hydrophobic interaction, the oil droplets adsorb on the surface of the new membranes (Figure 1a, left side). However, when the surface has been hydrophilized (Figure 1b), the oil adsorption is limited [79]. If the feed contains SDS, it covers both the surface of the oil droplets and the surface of the hydrophobic membrane which enables oil deposition (Figure 1a, right side). If a layer of water is adsorbed on the membranes surface, the adsorption of the oil is limited, which changes when the SDS forms a bilayer micelle (Figure 1b, right side).

Fast wetting of MD membranes during the separation of salt solutions containing high concentrations of surfactants (e.g., 10–25 mg/L [7]) has been widely documented in the literature [20,75,76]. However, most of the studies available in the literature were performed during relatively short operating times, not exceeding 50 h. It is important to note that small amounts of surfactants may be present in desalinated water contaminated by bilge waters discharged from ships [81]. In addition, as shown in Figure 1, the oil dispersed in the emulsion may sorb the surfactants, which also may have an effect on the feed interaction with the membranes surface. In view of the above conclusions, the aim of this study is to investigate the effect of low-concentrated (1.5–2.5 mg/L) surfactants’ presence in oil-contaminated high-salinity solutions on the MD process efficiency during long-term module operation. Since the cations present in the feed, especially Ca^2+^, also have a significant influence on the wetting of the membrane surface by surfactants [82], natural water from the Baltic Sea was used in the present study.

## 2. Materials and Methods

**Figure 2:**
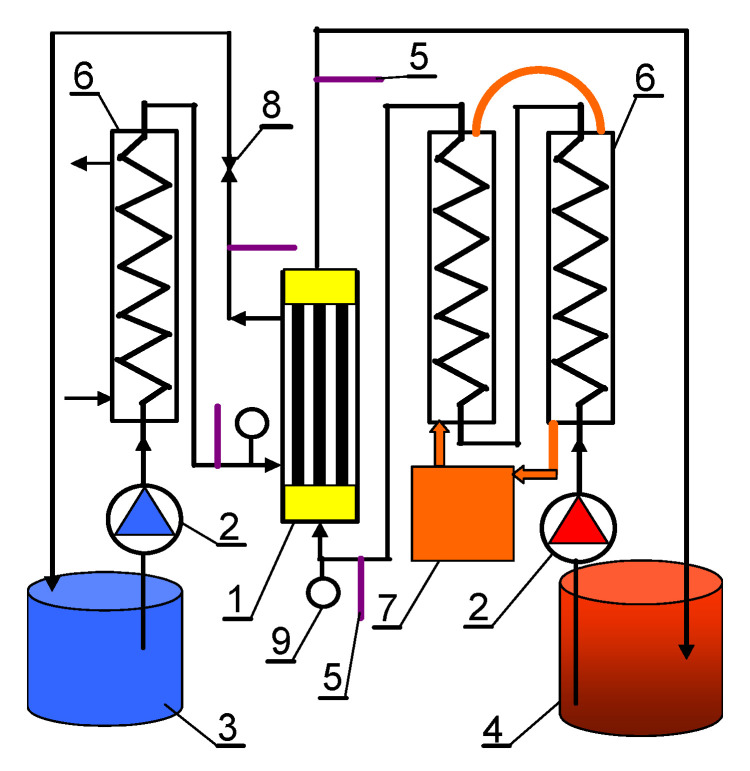
Schematic diagram of the DCMD installation. 1—capillary MD module, 2—peristaltic pump, 3—distillate tank, 4—feed tank, 5—thermometer, 6—glass spiral cooler, 7—thermostat, 8—valve, 9—manometer.

**Figure 3:**
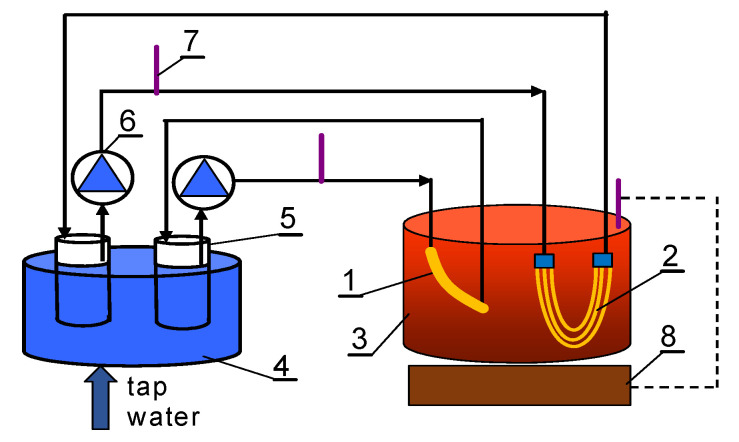
Schematic diagram of the MD installation with submerged modules. 1—submerged SMD-V8 module, 2—submerged SMD-S6 module, 3—feed tank, 4—cooling bath, 5—distillate tank, 6—peristaltic pump, 7—thermometer, 8—magnetic mixer with heating element.

Among various MD configurations, direct contact membrane distillation (DCMD) is the simplest and the most widely investigated for various industry solutions [15,18,83]. Indeed, it was recognized as the most favourable for the concentration of aqueous solutions in which water is the major permeating component [84]. Hence, in the present paper, in order to investigate the MD process, the DCMD configuration was applied. Importantly, increased transmembrane pressure (TMP), due to, e.g., the flow in the capillary module, may accelerate the wetting of the membranes pores compared to systems with the membranes submerged in the feed [85]. Therefore, in the present study, the wetting of membranes in capillary and submerged modules was compared. The immersion of membranes inside a non-pressure feed vessel allows elimination of hydrostatic pressure generated by feed pumping. The membranes were tested using two types of modules: the capillary module (CMD) placed in the installation shown in Figure 2 and the submerged module (SMD) without the outer casing in the installation presented in Figure 3.

For the capillary module construction, two types of Accurel PP membranes (Membrana GmbH, Wuppertal, Germany) were used: (1) Accurel PP S6/2 membranes with an internal diameter of 1.8 mm and a wall thickness equal to 0.4 mm, (2) Accurel PP V8/2 HF membranes with a diameter of 5.5 mm and a wall thickness of 1.5 mm. The membranes used were characterized by a porosity equal to about 70% and a nominal pore diameter of 0.2 µm (manufacturer data). The parameters of the modules used are presented in Table 1.

The CMD module consisted of three capillaries assembled together (Table 1). It was placed in a vertical position, the streams flowed co-current from bottom to top, and the feed was flowing inside the capillaries. The liquids were pumped by peristaltic pumps with a flow rate of 470 ± 5 mL/min, which allowed to obtain a linear flow velocity of the feed and distillate (space between capillaries) equal to 0.110 m/s and 0.054 m/s, respectively. Due to the smaller cross-section of the channel inside the capillaries, the feed pressure at the inlet of the module was 1560 Pa higher than the pressure on the distillate side. Experiments were also carried out for a similar pressure on both sides of the membranes, which was obtained by increasing the value of the distillate pressure by adjusting valve 8 (Figure 2).

The temperatures of the feed and the distillate were 343 ± 0.2 K and 292 ± 1 K, respectively. The feed volume was 2 L. In order to limit changes in the feed concentration, the permeate was periodically returned to the feed tank. The process performance was determined on the basis of the increase in the distillate volume, measured every 2 h. Subsequently, 1 L of water was left in the distillate tank, and the excess was returned to the feed tank.

During the process with the application of the submerged modules, the distillate was flowing (200 ± 5 mL/min) inside the capillaries. The feed was mixed (700 rpm) and heated by the magnetic stirrer connected to a thermometer (RCT type, IKA, Wilmington, NC, USA). The temperature of the feed was 343 ± 0.5 K. The resulting feed losses were periodically supplemented with distilled water. The cooling bath maintained the distillate temperature in the range of 291–293 K. The initial distillate volume in the tank was 1.5 L and its increment was measured every 24 h.

In the present study, in order to improve the understanding the role of low-concentrated (1.5–2.5 mg/L) surfactants’ presence in oil-contaminated brines in the membrane-wetting and -scaling phenomena, the following feed solutions were used:

(i) Baltic Sea water,
(ii) Baltic Sea contaminated by oil and SDS,
(iii) High salinity solutions (NaCl) contaminated by SDS and oil.

The Baltic Sea water, containing about 7 g/L of salt (Table 2), was collected in autumn in Dziwnów (Poland). As was been reported in [81], seawater may contain small amounts of oil discharged with the wastewater from ships. Hence, in the present study, machine oil collected from the bilge of a ship’s engine room was used as the oil. 5 mL of oil was added to 1 L of distilled water, and after mixing, was subjected to ultrasound treatment (620 W, Sonic-6D, POLSONIC, Warsaw, Poland) for 2 h. As a result, an oil-in-water emulsion concentrate was obtained, which was dosed to the feed solution. In turn, the use of the surfactant SDS (purity > 99%, Sigma Aldrich, Shanghai, China) was due to the fact that it is one of the most well-known ionic surfactants [68]. Finally, the high salinity solutions were prepared using deionised water and pure NaCl (a purity > 99%, ChemPur, Piekary Śląskie, Poland).

The oil concentration was determined by an oil analyser OCMA 500 (Horiba, Kyoto, Japan). The apparatus performs an infrared analysis after automatic extraction of the oil from the water sample with the solvent S316 (Horiba).

Conventionally, MD membrane wetting is detected by monitoring the distillate electrical conductivity [7]. Wetting the pores allows the feed to leak through the membrane, which in turn increases the salt concentration in the distillate. The curve showing the changes in conductivity over time allows to determine the type and intensity of wetting of the membrane wall [86]. In the present study, the electrical conductivity of the solutions was measured by a CPC-551 meter (Elmetron, Zabrze, Poland) which enabled automatic measurements at predefined intervals.

The membranes’ contact angle and the solutions’ surface tensions were measured with a Sigma 701 microbalance (KSV Instrument Ltd., Helsinki, Finland). The measurements were carried out using the Wilhelmy plate method at ambient temperature (293–294 K). The contact angles of the surfaces of the new membranes were 102.2° (S6) and 101.7° (V8). 

A Malvern Mastersizer 3000E (Malvern Instruments, Malvern, UK) apparatus was used to determine the oil droplet size distribution in the emulsions. It allowed to measure particle diameters in the range of 0.1–3000 µm. The values reported in the present study are the average values of three repeated measurements. Deionized water (0.05 μS/cm) was used as the dispersion phase. The Malvern laser diffraction technique generates a volume distribution for the analysed light energy data. The calculations were performed using the Mie theory recommended for spherical particles which completely solves the equations for interaction of light with matter.

The membrane morphology was observed via scanning electron microscopy SEM connected with energy-dispersive X-ray spectrometer (EDS) (Hitachi, Tokyo, Japan).

## 3. Results and Discussion

### 3.1. MD Process Efficiency

#### 3.1.1. The Capillary Module

**Figure 4:**
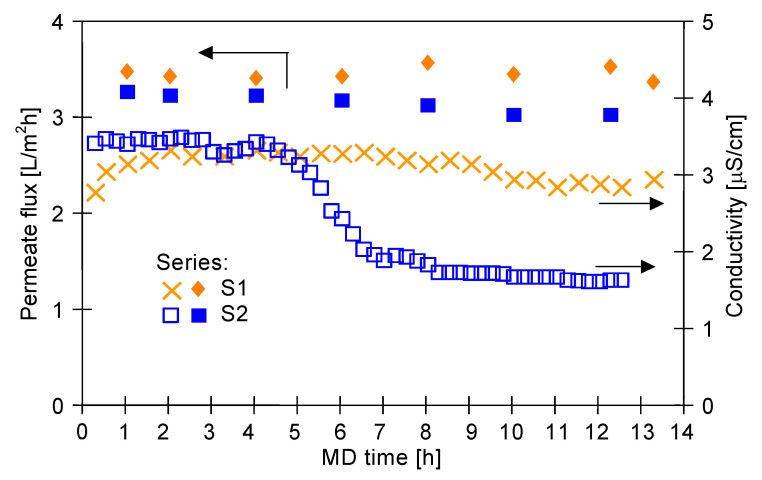
Changes in the permeate flux and distillate conductivity. Feed: Baltic Sea water (series S1), Baltic Sea water + oil (85 mg/L) + SDS (1.5 mg/L) (series S2). Module CMD.

In the initial stage of the MD process investigations, the capillary module CMD (Table 1) was used. The obtained results showed that Baltic Sea water characterized by a surface tension of 73 mN/m did not lead to wetting of the PP membranes. Indeed, the noted permeate flux was approximately constant and equal to about 3.4 L/m^2^h (Figure 4, series S1). Distillate conductivity of about 3 µS/cm was measured. The resistance of the PP membrane to wetting was also demonstrated in a previous study [25], in which the Baltic Sea water desalination process was carried out for over 2000 h. After completion of the presented run, the MD processing of the feed containing Baltic Sea water, oil (85 mg/L) and SDS (1.5 mg/L) was performed (series S2).

As stated before, the presence of low surface tension compounds in the feed may affect the feed and membrane surface characteristics. Therefore, it can be expected that the presence of SDS will lead to the membrane-wetting phenomenon (Figure 1). In the present study, it was found that presence of the SDS and oil lowered the feed surface tension rapidly, from 73 to 57.4 mN/m. Indeed, after one hour of the MD process run, a surface tension of 56.9 mN/m was recorded. However, although the feed was contaminated by oil and SDS, the membrane material still retained its non-wettability (Figure 4, series S2). This can be explained by the fact that the free energy of polypropylene surface is in the range of 28–35 mJ/m^2^ [21], hence, PP membranes are not wetted by liquids characterized by the surface tension above 30 mN/m [72]. This result was confirmed by the experiments conducted with the use of Accurel PP S6/2 membranes, which have a similar structure to the V8 membranes mounted in the CMD module. The module SMD-S6-0 was placed on tissue paper and Baltic Sea water contaminated with oil and SDS (57.1 mN/m) was compressed at 0.15 MPa (much higher than that applied in the MD modules). After an hour, no leakage through the walls of the membranes was found, which confirmed the non-wettability of the membranes by the test solution.

As expected, the presence of oil in the feed stream resulted in a noticeable reduction in the permeate flux. Indeed, a decrease in the flux from 3.4 to 3.1 L/m^2^h was reported. As shown in a previous study [79], this is presumably linked to oil adsorption on the membrane surface. The oil layer blocks the surface pores, which limits water evaporation and at the same time prevents feed leakage through the wetted pores, which significantly reduces the distillate conductivity to about 1.5 µS/cm. To confirm the oil adsorption on PP membranes, when the experiments were completed, the CMD module was rinsed with 20 mL of S316 solvent. Analysis of the oil content in the solvent sample (OCMA apparatus) demonstrated that 14.5 mg of oil had been adsorbed to the membrane surface in the CMD module.

**Figure 5:**
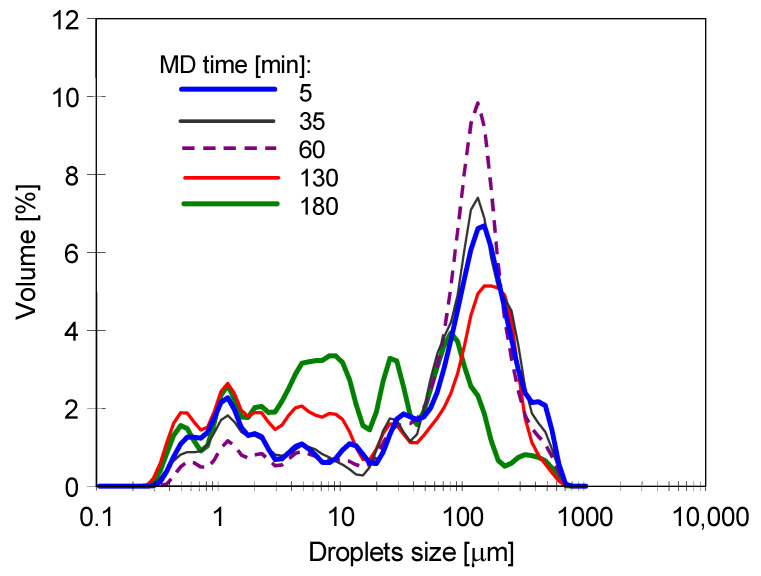
Changes in the droplet size distribution during the MD process. Feed: Baltic Sea water + oil (85 mg/L) + SDS (1.5 mg/L). Module CMD.

During the MD process, the feed flowed through the CMD module and returned to the feed tank. Importantly, a layer of separated oil formed on the feed surface in the tank. Probably, the feed flowing through the MD installation interacted with the membranes and pipes walls, leading to coalescence, that is, merging of droplets, and the emulsion breaking. This is consistent with the results of the droplet size distribution measurements (Figure 5). Indeed, it has been found that droplet sizes varied during the MD process. The emulsion is an unstable system, and the oil droplets naturally tend to stick together. As a result, smaller oil droplets are formed into larger ones, which allows them to rise to the liquid–air surface. As can be observed in Figure 5, at the beginning of the experiments, droplets with a size between 0.2 to 800 µm in the feed were present. It can be seen that during the first hour of the MD process, the number of observed large droplets (>100 µm) increased. After 2 h, the number of such droplets decreased, which confirmed their separation from the feed. Finally, after 3 h, mainly droplets smaller than 100 µm remained in the feed solution. It is generally assumed that the presence of surfactants in the system stabilizes the emulsion by preventing the oil droplets from merging [87]. Indeed, as has been mentioned earlier, surfactants may lead to reduced interfacial tension or the formation of a viscoelastic interfacial film. It is interesting to note that the experimental data obtained in the present study, do not confirm this observation. It has been found that low concentrations of SDS (1.5 mg/L) do not affect the above-mentioned phenomenon. This can be attributed to the fact that the hydrophobic interactions were strong enough to overcome any repulsive electrostatic or steric interactions leading to droplet aggregation occurs [88]. It should be noted that the surface area of the droplets increases as their diameter decreases, thus, more SDS particles are required to saturate the surface of the emulsion composed of smaller droplets.

**Figure 6:**
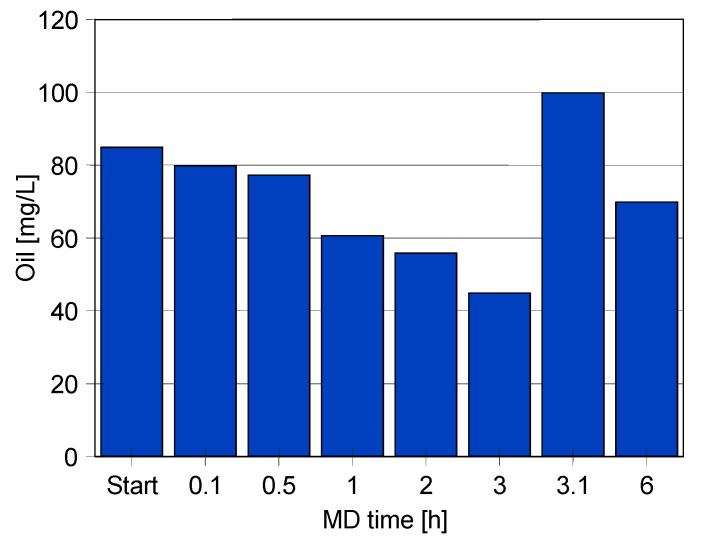
Oil concentration changes in the feed during the MD process of Baltic Sea water contaminated by oil and SDS (1.5 mg/L). Module CMD.

The analysis of the oil content in the feed confirmed oil separation and its accumulation on the liquid surface. As a result of the oil droplet separation, its concentration in the feed was decreasing, hence, a new portion of the emulsion was periodically added to the feed. Figure 6 shows the changes in oil concentration during the first 6 h of the MD process. It has been determined that during the first 3 h of the experiment’s run, the oil concentration decreased from 85 to 42 mg/L. Subsequently, a new portion of the oil was dosed (3.1 h), leading to an increase in the oil concentration to 100 mg/L. Finally, after 6 h of the process run, the oil concentration in the feed was equal to 64 mg/L. Additionally, the results have demonstrated that changes in the oil concentration affected the feed surface tension. Indeed, surface tension values of 64.1, 64.4 and 51.5 mN/m were recorded when the feed contained 36.2, 42 and 68 mg/L of oil, respectively.

**Figure 7:**
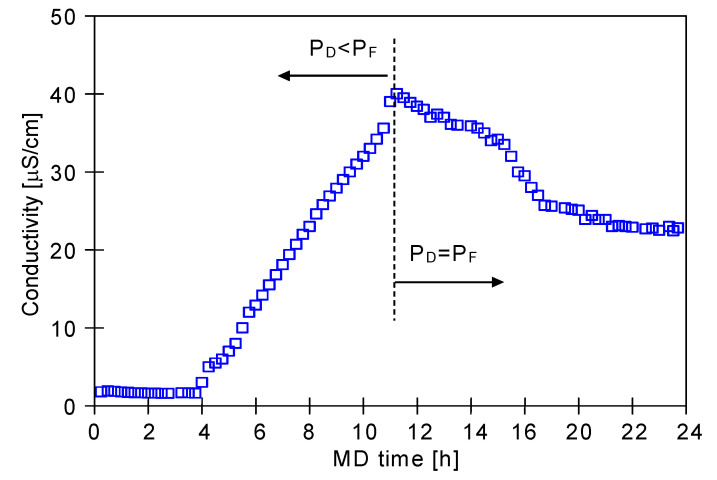
Changes in the distillate conductivity during the MD process. Feed: Baltic Sea water + oil (105 mg/L) + SDS (2.5 mg/L). Dashed line—the moment of equalizing the hydraulic pressure on the feed (P_F_) and distillate (P_D_) sides. Module CMD.

A key factor required for MD implementation at the pilot scale is that the feed solution does not wet the membranes during long-term operation. However, the results of long-term MD studies (i.e., process times of more than 1000 h) presented in the literature [33,79] have shown that for the initial 10–50 h of the process run, the membranes show resistance to wetting, after which increasing wetting of the pores was observed. Therefore, in the next stage of the research presented, the membranes’ wetting resistance for another 24 h was investigated. For this purpose, as a feed, Baltic Sea water contaminated with oil (105 mg/L) and SDS (2.5 mg/L) was used. It was found that although the feed was characterized by lower surface tension (42.4 mN/m) than in the above-presented process run, the distillate conductivity did not increase, remaining below 2 µS/cm for nearly 4 h of the experiment. It is worth noting that similar values were reported during the previously performed experiment series (Figure 4, series S2). However, in the following hours of the MD process, the conductivity steadily increased and after 11 h, a value of 40 µS/cm was noted (Figure 7). The feed conductivity was over 11,000 µS/cm; hence, it can be inferred that good wetting resistance of the membranes was still maintained and only a small proportion of pores had been wetted. This observation indicates the second degree of MD membranes wetting (partial wetting) that follows the first one (surface wetting) [89]. It is necessary to mention that the negative effect of partial wetting depends on the number of leakage channels formed through the membrane walls [86] and usually its intensity decreases, which allows the MD modules to be operated for thousands of hours [25].

In the experiments conducted, the volumetric flow rate of the feed and distillate was the same (470 mL/min). However, since the cross-section inside the capillaries was smaller, the pressure on the feed side was higher by 1560 Pa. Therefore, in the next stage, the pressures on both membrane sides were equalized, which allows to stop the leak if the partial wetting occurs. Consequently, the distillate conductivity was decreasing and finally, stabilized at the level of 28 µS/cm. Taking into account the relatively low value of conductivity and its stabilization in the last 5 h of the process run, it can be inferred that only a few pores larger than the dominant size (0.2 µm) were wetted. It is necessary to mention that throughout the process run, the permeate flux was stable and equal to about 3 L/m^2^h.

**Figure 8:**
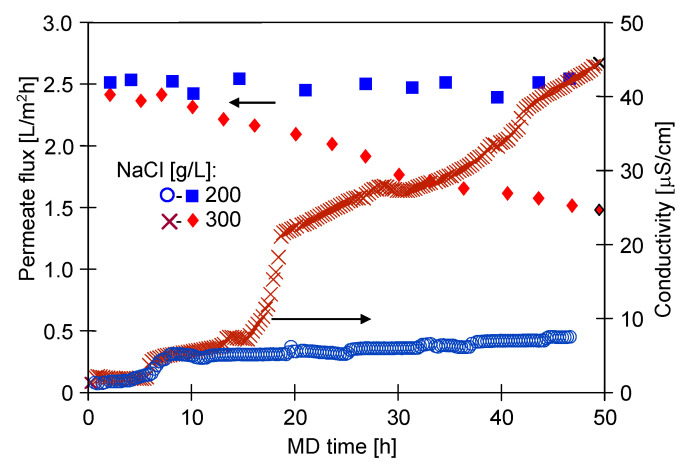
Changes in the permeate flux and distillate conductivity. Feed: NaCl solution (200 or 300 g/L) + oil (50–130 mg/L) + SDS (2 mg/L). Modules: CMD2 (200 g/L of NaCl) and CMD3 (300 g/L of NaCl).

During the separation of Baltic Sea water, the distillate obtained was periodically returned to the distillate tank, hence, the temporary concentration of the feed did not exceed 25%. However, in desalination installations, the aim is to obtain a very high water recovery rate, in the order of about 75–80%. For sea water (35 g/L), this will lead to an increase in the salt concentration to more than 150 g/L. Moreover, as mentioned above, if the water is contaminated with surfactants, the increasing salt concentration may significantly accelerate the membrane’s wetting. Therefore, in the next stage of the research presented, MD processing of concentrated NaCl solutions was carried out (Figure 8). For this purpose, the new CMD modules were used. The modules were preconditioned with a solution containing 1 g/L NaCl, which allowed to generate some additional hydrophilic groups on the membrane’s surface [79,80] which changes the course of the membrane-wetting phenomenon (Figure 1). During the initial 50 h of the series run, a stable permeate flux equal to about 3.5 L/m^2^h was obtained. Distillate conductivity in the range of 1.5–1.7 µS/cm was recorded. In the experiments conducted, the hydrostatic pressure on both sides of the membranes was balanced (P_F_ = P_D_).

Studies of the separation of contaminated high-salinity solutions were initially carried out with feeds containing NaCl (200 g/L), SDS (2 mg/L) and oil (113 mg/L). The surface tension of the solution was equal to 35.5 mN/m. The distillate conductivity increased slightly and after almost 50 h of the process run was equal to 8 μS/cm (Figure 8, series 200 g NaCl/L). This result indicates that despite the high concentration of NaCl in the feed (200 g/L), a small amount of SDS (2 mg/L) did not lead to wetting of the membranes used. It is worth emphasizing that after 45 h of the process, significant differences were noticed in the surface tension and oil concentration in the feed—the surface tension increased to 42.7 mN/m and the oil concentration decreased to 52.5 mg/L.

Remarkably, a more significant increase in the distillate conductivity was reported during the MD process of the feed containing salt (300 g/L), SDS (2 mg/L) and oil (130 mg/L) (Figure 8, series 300 g NaCl/L). The initial value of the feed surface tension was 33.6 mN/m and after 6 h it decreased to 30.1 mN/m with an oil concentration of 86.2 mg/L. The above-mentioned low value of the surface tension was probably the main reason for the sharp increase in the conductivity after 15 h of the process run. After 50 h of the MD process, a conductivity of 45 μS/cm was recorded. Considering several months of operation in an industrial installation, the reported continuous increase in the distillate conductivity clearly indicates that even low concentrations of SDS (2 mg/L) may accelerate the membrane-wetting phenomenon during brine separation. Moreover, a significant decline in the module performance was observed during the MD process of a feed containing 300 g/L of NaCl. These results suggest that the progressive wetting of the membranes caused decline in the permeate flux. This was also caused by the membranes scaling, which was discussed in the last Section of the manuscript (Section 3.2.1).

**Figure 9:**
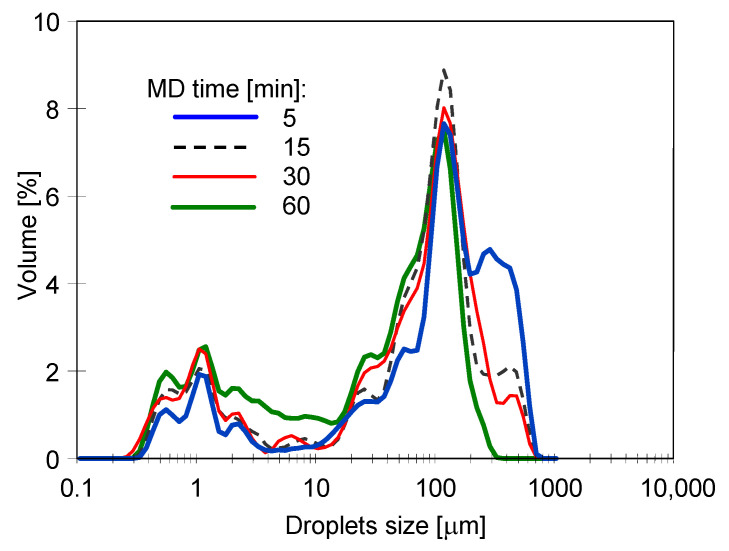
Changes in the droplet size distribution during the MD process. Feed: NaCl (200 g/L) + oil (85 mg/L) + oil (113 mg/L) + SDS (2 mg/L). Module: CMD.

The analysis of the droplet size distribution in the feed (Figure 9) showed that, similarly to the MD processing of Baltic Sea water, coalescence also occurred in the NaCl solution, as a result of which the large droplets formed were released on the feed surface. Moreover, it was observed that some of them adsorbed on the feed tank walls. Thus, as a result of coalescence, oil droplets with a dominant size below 100 µm were deposited in the feed. The small droplets do not form an extensive film of oil on the membrane’s surface and when they are compacted, their layers are separated by a thin film of water. It has been found that the tested membranes placed on such a layer did not wet during the many days of the experiments performed. In contrast, if large drops of oil were floating on the water surface, the membrane sample immediately saturated with it and sank.

#### 3.1.2. The Submerged Module

In the MD processing of NaCl solutions (200 and 300 g/L—Figure 8), the pressure on the feed and distillate sides was similar (2 kPa), which limited the feed leakage. Hydraulic pressure difference across the membrane module can accelerate the membrane wetting phenomenon [85]. However, maintaining equal pressure across the membrane module is a challenge and the results shown in Figure 7 indicate that in the flow modules, wetting even a small number of pores (partial wetting [89]) can cause significant leakage of feed into the distillate [86]. Hence, in order to avoid this issue, in the next stage of the present study, submerged modules with capillary distillate flow were used. In this case, the membranes are submerged in the feed tank and in order to increase the intensity of heat transfer and reduction in polarization phenomena, the feed tank can be equipped with a mixing system [90].

**Figure 10:**
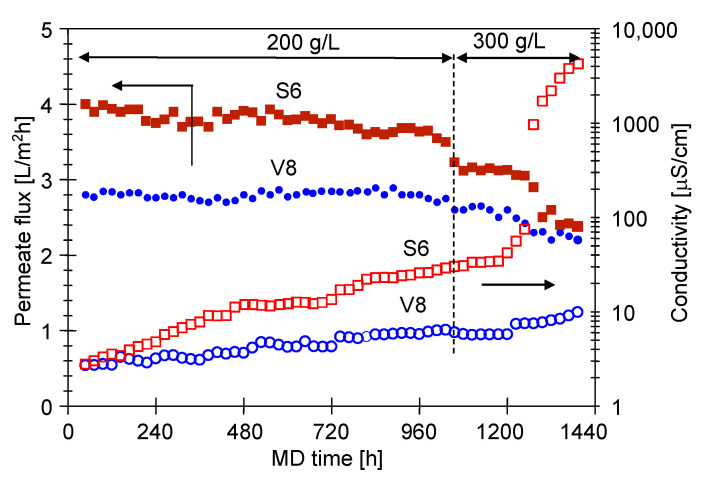
Changes in the permeate flux and distillate conductivity. Feed: NaCl solution (200 or 300 g/L) + oil (130–153 mg/L) + SDS (2 mg/L). Modules: SMD-S6-1 and SMD-V8-1.

As it has been pointed out by Madalosso et al. [15], membranes’ resistance to wetting during long-term operations is one of the most desirable membrane properties required for MD in industrial applications. Therefore, in the present study, the MD process with the use of submerged modules was carried out for a much longer time. Indeed, the module was operated continuously for 2 months (Figure 10). This allowed us to create the conditions presented in Figure 1b, which are more similar to those found in industrial installations. In addition to the Accurel PP V8/2 HF membranes, the experiments were conducted with the use of Accurel PP S6/2 membranes characterized by a wall four times thinner (Section 2). The investigations were performed with highly concentrated feed solutions. In the first stage (1080 h), the MD process was carried out for a feed containing salt (200 g/L), oil (130 mg/L) and SDS (2 mg/L). The surface tension of solution was 35.9 mN/m at the beginning of the process, 33.8 mN/m after 3 days and 43.8 mN/m after 45 days. After that, the feed tank was filled with a solution containing NaCl (300 g/L), SDS (2 mg/L) and oil (153 mg/L). The solution had a surface tension of 36.4 mN/m. Increasing the salt concentration accelerated the separation of oil from the feed and it has been found that after 3 days of the MD process, the oil concentration in the feed decreased to 68 mg/L. Subsequently, a portion of the oil was added to the feed and, consequently, its concentration increased to 144.6 mg/L. Finally, after 60 days of the MD process run, the surface tension of the feed was 33.7 mN/m.

Results obtained in the present study (Figure 10) have demonstrated that after 30 days of the modules operation (720 h), the efficiency of MD process changed significantly. Indeed, changes in the stabilized permeate flux values, for both the Accurel PP S6/2 and Accurel PP V8/2 HF membranes, were noted. In addition, slight changes in the distillate conductivity were observed. After another 13 days of the MD process run (Figure 10, 1050 h), it increased from 4.3 to 5.83 µS/cm and from 15.1 to 34.4 µS/cm, for the Accurel PP V8/2 HF and Accurel PP S6/2 membranes, respectively. Increasing the feed concentration from 200 to 300 g/L (from 1080 h) led to more significant changes in the MD process efficiency. Importantly, a particularly large increase in conductivity to 4100 µS/cm was observed for the Accurel PP S6/2. For the Accurel PP V8/2 HF membranes the conductivity increased to 10 µS/cm. Therefore, it can be concluded that the wall thickness of membranes has a great impact on their durability. Indeed, for the four-times-thinner membranes (Accurel PP S6/2), the pore wetting is much more significant. The obtained data indicated that after about 1300 h of the MD process, the partial wetting of S6 membranes wall increased abruptly, and then it stabilized [86]. 

**Figure 11:**
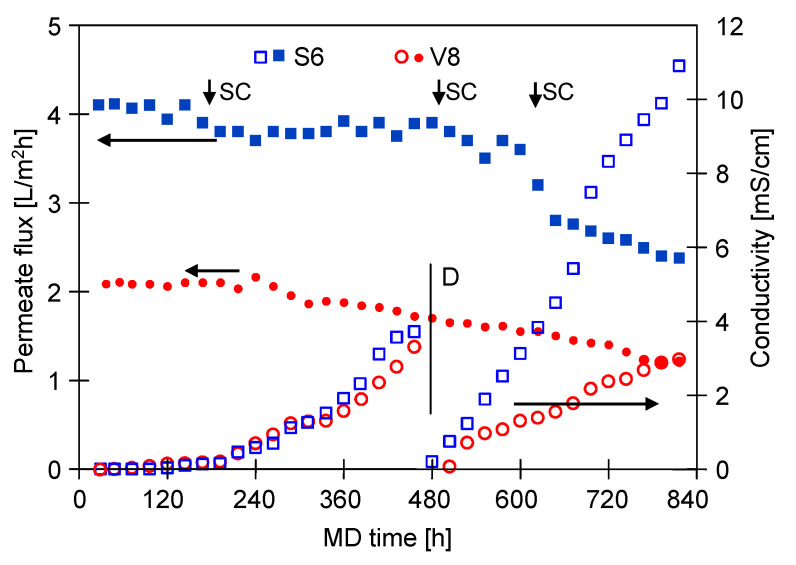
Changes in the permeate flux and distillate conductivity during continuous feed separation. Feed: NaCl solution (300 g/L) + oil (112–139 mg/L) + SDS (2 mg/L). Modules: SMD-S6-2 and SMD-V8-1. SC—temporary salt crystallization on the membrane surface; D—distillate tank filled with fresh distilled water.

Due to the significant S6 membrane wetting found in the experiment presented above, a new module (SMD-S6-2) was installed in the DCMD installation (Figure 3) and the investigation was resumed. It can be seen from the Figure 11 that the permeate flux (4 L/m^2^h) of the new S6 membranes, was higher than that reported in the previous MD run using the SMD-S6-1 module for a feed containing 300 g/L salt (Figure 10). This finding clearly indicates that the decrease in the permeate flux shown in Figure 10 was mainly due to the increasing intensity of the membrane wetting phenomenon. Moreover, for the initial 180 h of the process run (Figure 11), both for the new (SMD-S6-2) and previously operated (SMD-V8-1) modules, stable permeate flux was obtained and the distillate conductivity did not exceed 30 μS/cm. In turn, in the following hours, the membranes performance decreased slightly and a significant increase in the distillate conductivity was reported. The exponential increase in conductivity in the initial 480 h of the process run (Figure 11) indicates an increasing number of wetted pores [86]. It has been noted that for the V8 membrane, the exponential increase in conductivity was limited in the following hours, thus, it can be assumed that the wetting intensity of the membrane with a thicker wall was reduced.

It can be assumed that the above observations were due to the membrane scaling, discussed in detail in Section 3.2. In the DCMD process, as water vapor is transported through the membrane pores, the salt concentration at the interface between the membrane and the feed increases [53]. Therefore, as expected, collection of the permeate obtained during the MD process of a solution containing 300 g/L of NaCl resulted in a rapid increase in the salt concentration. In order to prevent saturation, portions of distilled water were systematically dosed into the feed tank, which allowed to maintain the salt concentration in the range of 290–315 g/L. However, three times (about 180, 480 and 600 h of the process run) the concentration of the feed increased to supersaturation and salt crystals were formed on the membrane’s surface, which dissolved after diluting the feed to a salt concentration of 290 g/L. Results shown in Figure 11 (points SC) demonstrate that the salt crystallization led to step changes in the permeate flux and an increase in the distillate conductivity. Importantly, these noteworthy results are in a good agreement with the work of McGaughey et al. [77] where the authors demonstrated that salt crystallization on the membrane’s surface (scaling phenomenon) significantly accelerates membrane wetting.

After a second salt crystallization in 480 h of the MD process run, the distillate was removed from the distillate tanks and the tanks were immediately filled with fresh distilled water (1.5 L) (Figure 11, point D). It has been noted that for the Accurel PP S6/2 membranes, the distillate conductivity increased faster and after the next 130 h it was about 4 mS/cm. This observation indicates that the subsequent salt crystallization (480 h) increased the intensity of the membrane wetting phenomenon. At the same time, For the Accurel PP V8/2 HF membranes, the rate of increase in distillate conductivity was similar to that noted in the previous stage of the process (160–480 h). Therefore, it can be concluded that membranes with a thicker wall show greater resistance to scaling.

As mentioned earlier, it is well known from the literature that the feed composition and, thus, the feed surface tension, may have a significant influence on the membrane phenomena. In the present study, it has been determined that the surface tensions of NaCl solutions containing 200 and 300 g/L of salt, were 71.3 mN/m and 79.4 mN/m, respectively. It is interesting to note that the above values changed significantly after adding the oil in the form of an emulsion (105 mg/L) to the feed solutions, becoming 57.7 mN/m (200 g/L) and 55.7 mN/m (300 g/L), respectively. Adding SDS (2 mg/L) to the solution containing 300 g/L of NaCl, led to a reduction in the surface tension to 33.8 mN/m. Undoubtedly, such a low value had an impact on the acceleration of pore wetting during the tests presented in Figure 11. As shown in Figure 1, in the case of separation of multiphase solutions, such as emulsions, the intensity of pore wetting may be influenced, apart from the surface tension, by the interaction of SDS with oil droplets [91] and the adsorption of the feed components on the membrane’s surface [71]. Accordingly, in order to investigate the impact of the above-mentioned phenomena on the MD process efficiency, in the last stage of the research presented, experiments with various compositions of the feed were carried out.

**Figure 12:**
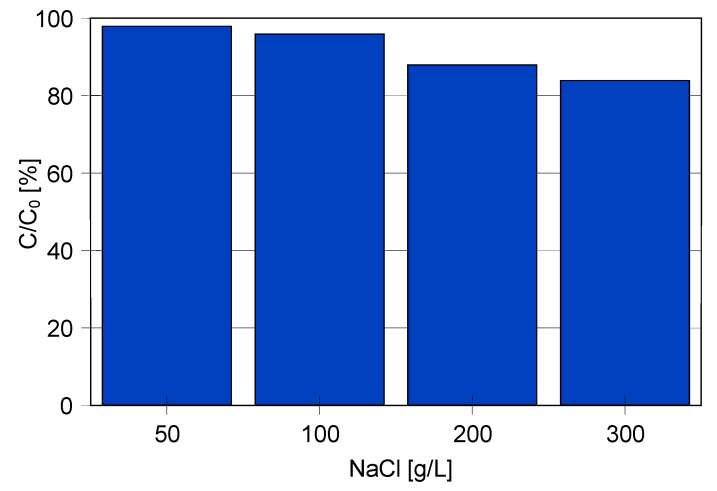
Changes in oil concentration after 3 h storage of solutions initially containing 100 mg/L of oil.

The concentration of salt in the emulsion has a significant influence on its stability. To the best of our knowledge, most of the work concerning oil separation using the MD process show results for a feed with a salinity corresponding to sea water (35 g/L) or slightly higher. Meanwhile, the results presented in Figure 12 indicate that concentrated solutions with a salt concentration of 200–300 g/L accelerate the separation of oil from the feed. 

**Figure 13:**
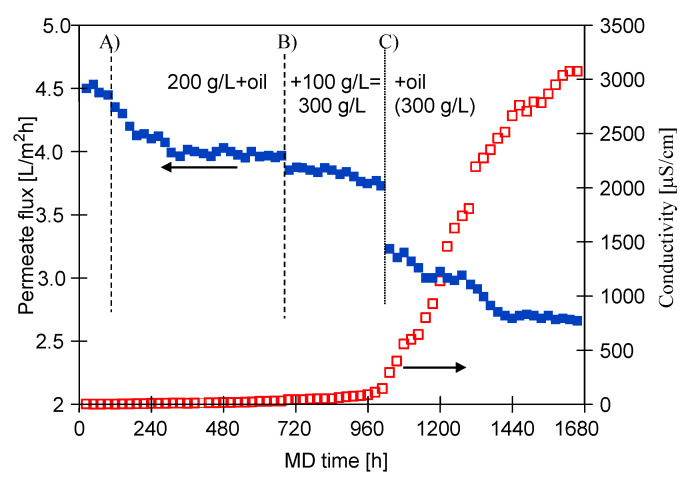
Changes in the permeate flux and distillate conductivity for various feed solutions. Initial feed: NaCl solution (200 g/L). Added to the feed: (A) oil 108 mg/L, (B) 100 g/L NaCl and (C) oil 112 mg/L. Module: SMD-S6-3.

In order to determine the effect of the oil, studies on the separation of concentrated NaCl solutions (without SDS) were carried out with periodic addition of the emulsion. The experiments were started by feeding the SMD-S6-3 module with the solution containing 200 g/L of salt. During the first 100 h the permeate flux was of 4.5 L/m^2^h (Figure 13). After this period, the emulsion concentrate was added to the feed, resulting in an oil concentration of 108 mg/L, which decreased to 65 mg/L during the next 3 days. Probably due to oil absorption, during the next 240 h permeate flux decreased from 4.5 L/m^2^h to 4 L/m^2^h and remained stable at that level during the next 300 h. At the end of this period, the distillate conductivity increased slightly to 38 μS/cm and the oil concentration was 32 mg/L. When the feed also contained oil and SDS, during the first 24 h of process, a permeate flux of about 4 L/m^2^h was noted and it was slightly lower (3.85 L/m^2^h) after 700 h of MD process (Figure 10). Therefore, it can be concluded that the presence of the SDS component led to an accelerated membrane wetting phenomenon. This observation can be related to several mechanisms identified in the literature. First of all, as described by Velioğlu et al. [92], NaCl can break the SDS–water network, leading to an increase in SDS affinity for the membrane and, consequently, to an increase in migration of SDS molecules to the membrane. This in turn can result in a reduction in the surface tension at the membrane pores, leading to an increased the likelihood of exceeding the liquid entry pressure. Secondly, according to Mahmoudvand et al. [93], the presence of salt ions may increase the surface activity of surfactants and, as a consequence, significantly reduce the feed surface tension.

After 700 h of the MD process, the salt concentration in the feed was increased from 200 to 300 g/L (Figure 13, point B), which caused a decrease in the permeate flux from 4 to 3.8 L/m^2^h and an increase in the distillate conductivity to 150 μS/cm. The feed contained only 30 mg/L of oil; hence, the increase in salt concentration did not cause a significant decline in the permeate flux. Nevertheless, a more significant reduction in the module efficiency was noted when an additional portion of oil emulsion (112 mg/L of oil) was added to the feed (Figure 13, point C). Initially, the permeate flux decreased to 3.25 L/m^2^h, which probably resulted from the adsorption of oil, the concentration of which decreased to 78 mg/L over 4 h. During next 500 h of the process run, the permeate flux stabilized at 2.7 L/m^2^h, while the conductivity increased to 3100 μS/cm. Therefore, results obtained in the present study demonstrate that the addition of oil to the concentrated brine (300 g/L) contributed to the membrane wetting. It was also found (Figure 10) that when the feed (300 g/L of salt) also contained SDS, the decrease in the permeate flux was more significant (about 2.25 L/m^2^h) and the conductivity reached 4100 μS/cm. Performing this comparison demonstrated that the presence of even a low concentration of SDS (2 mg/L) has a significant effect on membrane wetting.

### 3.2. Membranes Scaling

#### 3.2.1. The Capillary Module

In general, since the water feed in the MD process is highly saline and contaminated, inorganic fouling, commonly known as scaling [4], is considered to be one of the major problems in MD modules [53]. In the present study, in order to investigate the membrane scaling, SEM observations of membranes’ morphology and analysis of their surface composition were carried out. It was determined that during the conducted experimental studies, a deposit layer containing salts was formed on the membranes’ surface. What becomes apparent from these results, is that the increase in the distillate conductivity observed during the MD processes could be caused not only by the SDS present in the feed, but also by the membrane scaling phenomenon. Undoubtedly, this finding is consistent with results presented in Figure 11, wherein the reduction in the module’s efficiency due to the salt crystallization was demonstrated. In addition, it should be noted that, unlike the NaCl solutions, natural waters (e.g., Baltic Sea water) also contain other cations. In this case, the DS^−^ molecules and Ca^2+^ ions react to form calcium dodecyl sulfate, Ca(DS)_2_, which has low solubility and thus precipitates [94]. The formed deposit, Ca(DS)_2_, may also have an effect on membrane wetting.

**Figure 14:**
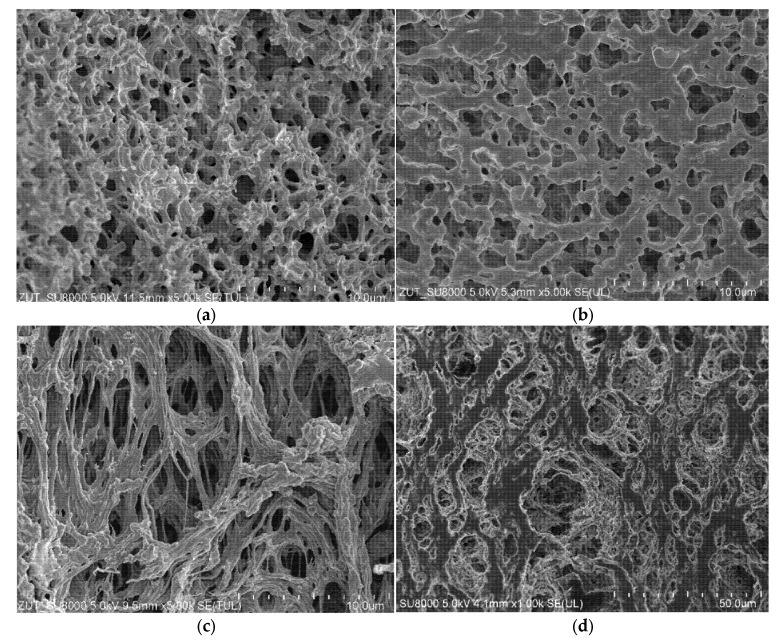
SEM images of the membranes used. The Accurel PP V8/2 HF membrane: (**a**) wall cross-section; (**b**) internal surface; (**c**) external surface. The Accurel PP S6/2 membrane: (**d**) external surface.

It was determined that the Accurel PP V8/2 HF membranes have walls characterized by a symmetrical sponge structure (Figure 14a). Furthermore, it has been found (Figure 14b) that the pores on the inner surface were smaller and characterized by higher level of size uniformity compared to those on the outer surface. A similar pore structure, both inside the wall and on the inner surface, was observed for the Accurel PP S6/2 membranes. In turn, on the capillaries outer surface of both membrane types, a layer 10–20 µm thick was observed, with significantly larger pores (Figure 14c), especially for the Accurel PP S6/2 membrane (Figure 14d). To be complete, it should be noted that such large pore sizes can facilitate the wetting phenomenon, especially for the submerged modules used, where the feed was flowing outside the capillaries.

**Figure 15:**
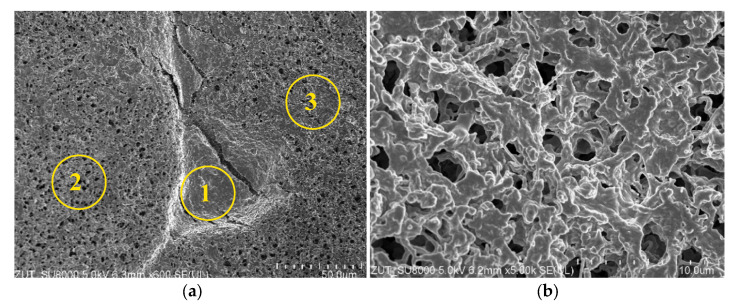
SEM images of the membrane’s surface after completing the MD studies: (**a**) feed: Baltic Sea water, CMD1 module; (**b**) feed: NaCl solution (300 g/L), CMD 3 module.

The SEM observations demonstrated that during the separation of Baltic Sea water, a deposit formed on the membranes surface. In several places, more extensive agglomerates of the deposit covering the pores were observed (Figure 15a—area 1). The results of the SEM-EDS analysis (Table 3) clearly showed the presence of significant amounts of Si, S and Ca in this deposit. This result indicated that the deposit could be formed mainly by silicates and calcium sulfate. Since SDS was also present in the feed, after dissociation it could react with Ca^2+^ ions to form insoluble Ca(DS)_2_ [94]. In addition, analysis of the surface areas without the large agglomerates (Figure 15a—areas 2 and 3) showed that small amounts of deposit were dispersed over the entire membrane’s surface. It should be pointed out that significantly larger amounts of deposit were formed when the CMD3 module was fed with the NaCl solution (300 g/L) (Figure 15b). The results of SEM-EDS analysis confirmed that the deposit contained NaCl. However, it did not have a crystalline structure, which most probably resulted from the presence of the organic pollutants (oil and SDS). Although the deposit did not cover the entire membranes surface, compared to the pristine membrane (Figure 14b), the surface pores were partially blocked, which was one of the reasons for the decrease in the process performance shown in Figure 8.

#### 3.2.2. The Submerged Module

**Figure 16:**
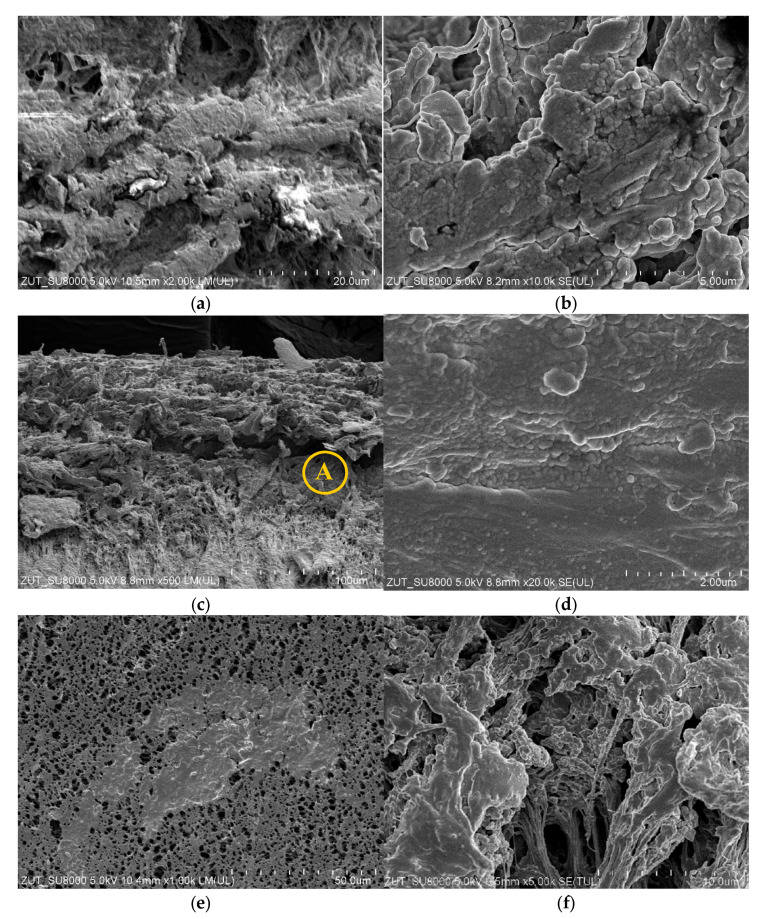
SEM images of the membranes used. Module SMD-S6-1: (**a**) external surface; (**b**) magnified (×10k) external surface; (**c**) cross-section; (**d**) the area A shown in (**c**) magnified (×20k); (**e**) internal surface. Module SMD-V8: (**f**) external surface.

Comparing the two types of membrane modules used in the present work, it was found that during the MD process of NaCl solution (300 g/L), more deposit was formed on the surface of membranes used in submerged modules. This is clearly related to a much longer service life of these modules. The results of the SEM observations indicated that the deposit covered the membranes surface unevenly and areas with open pores were found (Figure 16a). Similar to the findings for the CMD3 module presented in the previous Section 3.2.1, the deposit did not have a crystalline structure and the presence of spherical forms (Figure 16b) could result from oil droplets fixed in the salt deposit.

The SEM observations performed for the membranes’ cross-sections indicated that the deposit was formed not only on the membranes’ surface but also inside their walls (Figure 16c). It was found that due to the deposit build-up, the outer edge of the capillary was damaged to a depth of about 100 µm. The magnification of this area shown in Figure 16d demonstrates that the deposit in this area completely filled the membrane’s pores. Indeed, no walls of the polymeric pore inside this deposit were found. Moreover, newly formed spherical formations were observed. Such an intensive scaling phenomenon limited the evaporation surface, which resulted in a rapid decrease in the performance and effectiveness of the SMD-S6-1 module (Figure 10). It should be pointed out that in some membranes parts, the pores were wetted through the entire thickness of the wall, which led to the leakage of the feed and, as a result, a rapid increase in the distillate conductivity was noted. During drying of membrane samples, the feed flowed from the wetted pores forming a deposit on the distillate side (Figure 16e). The SEM studies confirmed that the deposit was also formed on the Accurel PP V8/2 HF membrane′s surface (Figure 16f), however, it did not form such a compact structure. This observation clearly explains the lower decline in the membrane performance noted for the SMD-V8 module (Figure 10, V8). Comparing the above results to those presented in the previous work [86], it can be inferred that the presence of organic pollutants, such as oil and SDS, significantly intensifies the internal scaling phenomenon.

**Figure 17:**
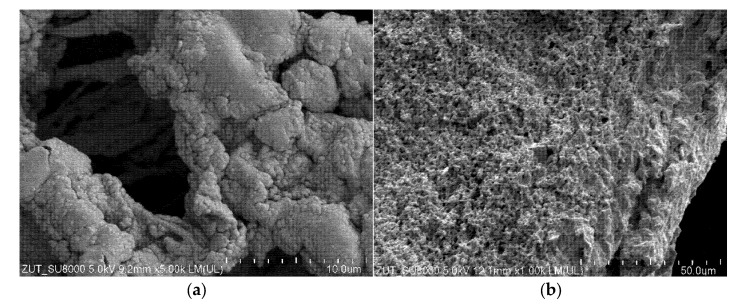

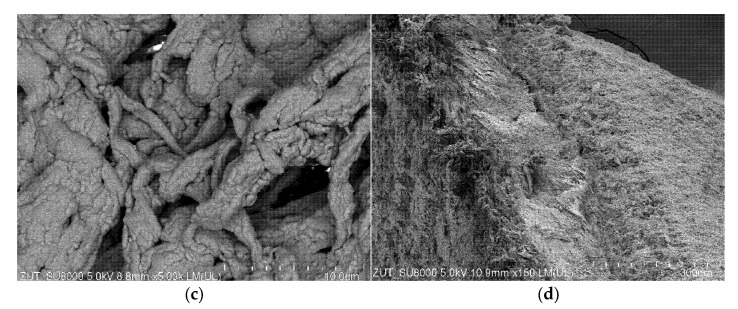
SEM images of the membranes used. Module SMD-S6-2: (**a**) external surface; (**b**) wall cross-section. Module SMD-V8: (**c**) external surface; (**d**) wall cross-section (external edge).

In the next stage of the research presented, the investigations were performed with a new SMD-S6-2 module. The membranes wetting resistance was determined by systematically measuring the distillate conductivity (Figure 11). It should be pointed out that the obtained conductivity values were higher than those noted during the operation of the SMD-S6-1 module. The results point to the likelihood that the feed was concentrated to a supersaturated state and, as a result of salt crystallization, the scaling phenomenon was more intense. Importantly, the SEM studies confirmed that large amounts of deposit were formed on the Accurel PP S6/2 membranes surface (Figure 17a). Observations of the wall cross-section indicated intensive internal scaling, with damage to the surface layer (Figure 17b) that promoted feed leakage, which in turn led to an increase in the distillate conductivity. It is necessary to mention that almost complete coverage of the membrane surface with deposit was also observed for the SMD-V8 module (Figure 17c). Similar to the results discussed above, obtained for the other membranes, deposit was formed as rounded/spherical forms. The wall cross-section observations showed that also in the SMD-V8 module, flowing of the feed into the membrane pores, led to the internal scaling phenomenon. Consequently, significant damage to the outer layer (over 200 µm) was observed and the thickness of the wetted layer exceeded 300 µm (Figure 17d). Surprisingly, although the membrane wall thickness was 1500 µm, such significant damage did not lead to significant feed leakage. Indeed, the noted distillate conductivity only increased to 154 µS/cm. These results indicate, however, that if the desalinated water contains even small amounts of oil and SDS, the use of the MD process to implement the concept of “Zero Liquid Discharge (ZLD)”, as a result of intense internal scaling, will encounter significant problems with the mechanical stability of the membranes. 

**Figure 18:**
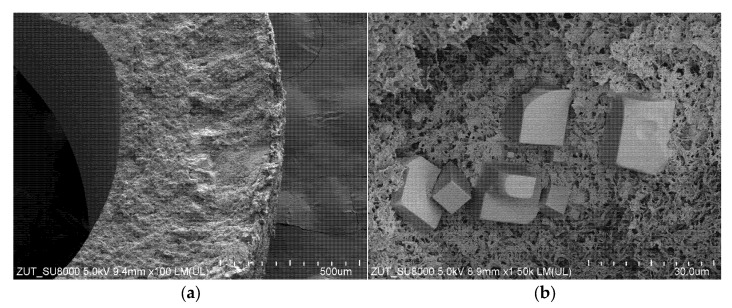
SEM images of the membranes used for the separation of NaCl solution (300 g/L) contaminated with oil (87–112 mg/L): (**a**) membrane cross-section; (**b**) NaCl crystals on the membrane surface. Module: SMD-S6-3.

In order to demonstrate and emphasize the effect of SDS on the membrane scaling phenomenon, the experiments were repeated with a saturated NaCl solution contaminated only with oil (87–112 mg/L). The MD process was carried out for nearly 1700 h. The SEM observations confirmed that also in this case a significant amount of deposit was formed on the membranes surface. However, the damage to the membranes in the SMD-S6-3 module was significantly less (Figure 18a) than in previously discussed MD processes where the feed also contained SDS. Moreover, the presence of NaCl crystals was found both in the deposit on the membrane’s surface and inside the wall (Figure 18b). These noteworthy results clearly demonstrate that the amorphousness of the formed deposits was mainly due to the presence of SDS.

## 4. Conclusions

Promising results with long-term studies have confirmed the good wetting resistance of Accurel PP membranes during the MD process of high-salinity solutions. It has been shown that the MD process can be successfully used for treating high-salinity solutions. Moreover, it has been shown that MD can also be successfully applied for desalination of brines contaminated with oil (up to 100 mg/L). The oil partially adsorbed on the membranes surface which resulted in reducing the process performance and partial wetting of the membrane’s pores. Nevertheless, even for solutions with a NaCl concentration of 300 g/L, salt retention of 99% was noted. However, as a result of salt crystallization on the membranes surface, the intense scaling phenomenon inside the wall led to membrane damage. As a consequence, leakage of the feed into the distillate was noticed. Therefore, in order to limit this unfavorable effect, it is recommended that the NaCl concentration of the feed to should be much lower than 300 g/L. It was demonstrated that increasing the membrane’s wall thickness from 0.4 to 1.5 mm resulted in a twofold reduction in the MD process performance and greater resistance to degradation. In addition, the obtained results indicate that the use of PP membranes for separation of feeds containing oil and SDS in the membrane distillation crystalizer is not possible due to the membrane’s destruction caused by the internal scaling phenomenon. It was demonstrated that the presence of SDS (1.5–2.5 mg/L) in the oil-contaminated high-salinity solution slightly accelerated the membranes wetting. This observation clearly indicates that the short-term presence of surfactants in the feed water should not affect the performance of the MD plant. However, in the case of installations permanently operated for the treatment of water contaminated with surfactants, it is advisable to use modified membranes characterized by high wetting resistance. To sum up, the overall findings presented in this study add substantially to understanding the effect of low-concentration SDS on MD process efficiency.

## Figures and Tables

**Table 1:** Characteristics of the capillary MD modules.

Module	Membrane	No. of Capillaries	Length(m)	Area (Inside) (m^2^)	Housing Diameter (m)
CMD	Accurel PP V8/2 HF	3	0.095	49.2 × 10^–3^	0.02
SMD-V8	Accurel PP V8/2 HF	1	0.25	43.2 × 10^–3^	not applicable
SMD-S6	Accurel PP S6/2	3	0.25	42.4 × 10^–3^	not applicable

**Table 2:** The ions concentration in the Baltic Sea water.

Ion	Na^+^	Cl^−^	SO_4_^2−^	Mg^2+^	Ca^2+^	K^+^	PO_4_^3−^
Concentration (mg/L)	2329	3720	538	504	137	78	7.5

**Table 3:** Concentration (wt.%) of elements forming the deposit.

Area	Si	S	Cl	Ca	K	Na	C
1	26.40	9.00	30.60	14.70	1.20	2.10	16.00
2	0.26	0.10	0.07	0.28	0	0.03	99.26
3	2.90	0.80	2.30	0.50	0.60	1.30	91.60

## Data Availability

The data presented in this study are available on request from the corresponding author. The data are not publicly available due to the institutional repository being under construction.
